# Detection Rate of ^68^Ga-PSMA Ligand PET/CT in Patients with Recurrent Prostate Cancer and Androgen Deprivation Therapy

**DOI:** 10.3390/biomedicines8110511

**Published:** 2020-11-18

**Authors:** Joachim Brumberg, Melanie Beckl, Alexander Dierks, Andreas Schirbel, Markus Krebs, Andreas Buck, Hubert Kübler, Constantin Lapa, Anna Katharina Seitz

**Affiliations:** 1 Department of Nuclear Medicine, University Hospital Würzburg and Julius-Maximilian University Würzburg, 97080 Würzburg, Germany; melanie@privat.beckl.de (M.B.); alexander.dierks@uk-augsburg.de (A.D.); Schirbel_A@ukw.de (A.S.); Buck_A@ukw.de (A.B.); Constantin.Lapa@uk.augsburg.de (C.L.); 2 Department of Urology, University Hospital Würzburg and Julius-Maximilian University Würzburg, 97080 Würzburg, Germany; Krebs_M@ukw.de (M.K.); Kuebler_H@ukw.de (H.K.); Seitz_A3@ukw.de (A.K.S.); 3 Comprehensive Cancer Center Mainfranken, University Hospital Würzburg and Julius-Maximilian University Würzburg, 97080 Würzburg, Germany; 4 Department of Nuclear Medicine, Medical Faculty, University of Augsburg, 86156 Augsburg, Germany

**Keywords:** ^68^Ga-PSMA ligand PET/CT, androgen deprivation therapy, detection rate, recurrent prostate cancer

## Abstract

Prostate-specific membrane antigen (PSMA) ligand PET/CT enables the localization of tumor lesions in patients with recurrent prostate cancer, but it is unclear whether androgen deprivation therapy (ADT) influences diagnostic accuracy. The aim of this study was to evaluate the effect of ADT on the detection rate of ^68^Ga-PSMA ligand PET/CT. Thus, 399 patients with initial radical prostatectomy and ^68^Ga-PSMA ligand PET/CT during PSA relapse were retrospectively evaluated. Propensity score matching was used to create two balanced groups of 62 subjects who either did or did not receive ADT within six months before imaging. All ^68^Ga-PSMA ligand PET/CT were evaluated visually and with semiquantitative measures. The detection rate of tumor recurrence was significantly higher in the group with ADT (88.7% vs. 72.6%, *p* = 0.02) and improved with increasing PSA-levels in both groups. In subjects with pathological PET/CT and ADT, whole-body total lesion PSMA (*p* < 0.01) and PSMA-derived tumor volume (*p* < 0.01) were significantly higher than in those without ADT. More PSMA-positive lesions and higher PSMA-derived volumetric parameters in patients with ADT suggest that a better detection rate is related to a (biologically) more advanced disease stage. Due to high detection rates in patients with PSA-levels < 2 ng/mL, the withdrawal of ADT before PSMA ligand PET/CT cannot be recommended.

## 1. Introduction

Biochemical recurrence (BCR), defined by an increase of the prostate-specific antigen (PSA), occurs in up to 50% of the patients within five years after radical prostatectomy (RP) [1]. Treatment decisions following BCR must balance the risk of metastatic disease to potential treatment side effects (e.g., on patients’ quality of life) while avoiding overtreatment at low risk of clinical progression. Different treatment modalities for managing these patients are available, but their application and timing remain controversial due to limited evidence [2]. Androgen deprivation therapy (ADT) is the standard treatment of locally advanced (in combination with radiation therapy) and metastatic disease [3,4]. In the BCR-setting, ADT should be reserved for patients at highest risk of disease progression, mainly defined by a short PSA doubling time (<6–12 months) [5]. However, BCR, continuously increasing PSA levels in patients with and without ADT, and a PSA relapse over time after an initial treatment effect in patients receiving ADT require effective staging procedures for recurrent prostate cancer (PC).

Within the past years, molecular imaging probes targeting the prostate-specific membrane antigen (PSMA) have established for PET/CT imaging in the primary and secondary staging of PC [5,6]. PSMA is a membrane protein, which shows increased expression levels in PC cells [7]. Moreover, ^68^Ga- or ^18^F-labeled PSMA ligands enable the localization of tumor manifestations and thus help to differentiate between local, regional, and systemic disease in recurrent PC, which is crucial for subsequent treatment decisions. A meta-analysis of retrospective studies and a recent prospective multicenter trial on ^68^Ga-PSMA ligand PET/CT revealed that detection rate in recurrent PC depends on the PSA-value, and rises from 38% (PSA-levels < 0.5 ng/mL) up to 97% (PSA-levels ≥ 5 ng/mL) [8,9]. However, the influence of androgen deprivation therapy (ADT) on the detection efficacy of PSMA ligand PET/CT is unclear since incongruent findings have been reported from different centers [10,11]. An association of a positive ^68^Ga-PSMA ligand PET/CT with ADT was observed, though the significance in multivariate logistic regression analysis depended on the inclusion and scaling of other variables [12]. Direct comparisons of the detection rate in patients with vs. patients without ADT are limited due to unmatched PSA-values between groups [13,14]. Furthermore, the actual biological effect of androgen receptor inhibition on PSMA ligand uptake is controversial. Whereas short-term ADT seems to increase the uptake at least in some PC lesions [15,16,17,18], the treatment effect of long-term ADT might be accompanied by a lower PSMA ligand uptake and thus, a reduced visibility of tumor lesions [19].

The aim of this study was therefore to directly compare the detection rate of ^68^Ga-PSMA ligand PET/CT in two balanced patient groups with and without ADT. We additionally estimated the tumor burden and PSMA expression by collecting ^68^Ga-PSMA ligand PET-derived quantitative parameters to disentangle whether deviant detection rates between groups is related to a higher visibility of tumor lesions or a different disease stage. Finally, this study aims to provide a recommendation for clinicians, who consider the withdrawal of ADT before PSMA-ligand PET/CT imaging.

## 2. Experimental Section

### 2.1. Study Design

This is a retrospective case-control study of patients with recurrent PC who underwent ^68^Ga-PSMA ligand PET/CT for diagnostic purposes between April 2014 and September 2018 at the Department of Nuclear Medicine, University Hospital Würzburg, Germany.

### 2.2. Patients

We extracted 572 patients with PSA relapse after initial therapy with curative intent, who performed ^68^Ga-PSMA ligand PET/CT from the institution’s database. Only patients who fulfilled the following criteria were included in the study: initial treatment with radical prostatectomy, a PSA-value between 0.2 and 20 ng/mL (not older than 3 months prior to imaging) and an available Gleason score from prostatectomy specimens. Patients with prior chemotherapy or new-generation androgen receptor inhibitor were excluded (for patient flow see Supplementary Figure S1).

### 2.3. Grouping

The patients (n = 399) were split into two groups, one containing patients, who received ADT during/within six months prior imaging (n = 65) [13], the second containing subjects without ADT (n = 334). The latter served to create a control group of patients without ADT, that was balanced to the group of patients with ADT. We used propensity score matching (caliper set at 0.05) for the assignment into the control group with following covariates: PSA-levels (ng/mL) converted to a natural logarithmic scale (log PSA), Gleason score, adjuvant or salvage radiotherapy and injected activity divided by body weight (MB q/kg).

### 2.4. Preparation of ^68^Ga-PSMA ligand

^68^Ga-PSMA imaging and therapy (I&T) labelling was performed on a fully automated GRP module (Scintomics, Fürstenfeldbruck, Germany) in combination with cassettes produced by ABX (ABX, Radeberg, Germany). A ^68^Ge/^68^Ga-generator (GalliaPharm^®^, Eckert & Ziegler AG, Berlin, Germany) was eluted with 0.1 N HCl, the eluate trapped on a cation exchange cartridge, and eluted with 5 N NaCl. Labelling of 20 µg PSMA I&T (Scintomics, Fürstenfeldbruck, Germany) was performed by heating the eluate for 6 min at 125 °C in the presence of HEPES-buffer. Then the reaction mixture was purified on a SepPak C18-cartridge, eluted with ethanol/water 50/50 and diluted with phosphate buffer solution to a total volume of 15 mL. A sample was taken for determination of radiochemical purity, radionuclide purity, ethanol content, pH, sterility, and endotoxins.

### 2.5. Imaging

All patients underwent ^68^Ga-PSMA I&T imaging on an integrated PET/CT scanner (Siemens Biograph mCT 64, Siemens Healthineers, Knoxville, TN, USA). The protocol included a distribution period of 60 min after injection of ^68^Ga-PSMA I&T. Whole-body PET was acquired in 3D-mode from the proximal thighs up to the upper jaw by using 2 min scan time per bed position. Subsequently, all patients underwent diagnostic CT scans with contrast enhancement and following parameters: 180 mAs (quality reference for dose modulation), 120 kV, 512 × 512 matrix, 5 mm slice thickness, 30 mm/s increment, 0.5 s rotation time, and a pitch index of 1.4. Three iterations, 24 subsets, and post-reconstruction Gaussian filtering of 2.0 mm full-width at half-maximum were used for iterative PET data reconstruction with attenuation correction (HD. PET, Siemens Esoft, Siemens Healthineers, Erlangen, Germany).

### 2.6. Image Analysis

All images were interpreted by one board-certified nuclear medicine physician (J.B.) and one board-certified radiologist (A.D.) in consensus reading on a syngo.via workstation (Siemens Healthineers, Erlangen, Germany). Then, ^68^Ga-PSMA I&T PET and CT images were rated for lesions suggestive for tumor recurrence and number of lesions for local recurrence, lymph node metastases (separated into pelvic, retroperitoneal, and supradiaphragmatic location), bone metastases and organ metastases were noted. PET images were assessed regarding focal uptake of ^68^Ga-PSMA I&T higher than the surrounding background and not related to a physiologic pattern [13]. In CT, any lymph node with a short axis diameter > 5 mm, pathologic pelvic lesions, and sclerotic bone lesions without association to degenerative changes were rated suggestive for malignancy. In all suspected pathological lesions, following PET-derived parameters were measured: highest maximum standardized uptake values (SUV_max_), total lesion PSMA (TL-PSMA), and PSMA total volume (PSMA-TV) [20,21]. Subsequently, we identified the highest SUV_max_ and summarized whole-body TL-PSMA and PSMA-TV (wbTL-PSMA, wbPSMA-TV) for each patient.

### 2.7. Statistical Analysis

The balancing of covariates after propensity score matching was evaluated by using students t and Chi-square test. The latter was also used to compare detection rate between groups. Binary logistic regression analysis was performed with the detection rate as dependent variable and the factors ADT (yes/no), radiotherapy (yes/no), Gleason score (5–7/8–10), log PSA, and injected radioactivity per kg body weight. Numeric and volumetric imaging-derived parameters were compared using Mann–Whitney U test. The association between PSA-level and imaging parameters was assessed with Spearman’s rank correlation coefficient (*σ*). Our main hypothesis was that the detection rate differs between groups, whereas the evaluation of imaging-derived parameters was considered exploratory. Therefore, we defined a *p* value < 0.05 as significant for all analysis. Quantitative values are displayed as mean ± standard deviation or as median and range, as appropriate. Data were analyzed with SPSS statistics (Version 25.0, IBM corp., Armonk, NY, USA).

### 2.8. Ethics Approval and Patient Consent

The retrospective analysis was approved by the local institutional review board of the Julius-Maximilian University Würzburg (AZ: 20200210-02; approval date: 27 February 2020). ^68^Ga-PSMA I&T PET/CT scans were performed as part of the clinical work-up and were in accordance with the principles of the Declaration of Helsinki and its later amendments. All patients gave written informed consent to the diagnostic procedure.

## 3. Results

### 3.1. Patient Groups

Before propensity score matching, the patients with ADT showed a significantly higher PSA-value, had a higher Gleason score, and were more often treated with radiation therapy than patients without ADT; the mean injected radioactivity did not differ between groups. Propensity score matching balanced all four covariates between groups by yielding a match for 62 of 65 patients with ADT. The remaining three patients were excluded. The covariates of both groups before and after matching are listed in Table 1, the demographic and clinical characteristics of the final case and control groups are shown in Table 2. By clinical definition [5] and considering PET/CT findings, 42 patients of the final ADT group had a hormone-sensitive PC (HSPC), whereas 20 patients had a castration-resistant PC (CRPC) at imaging.

### 3.2. Detection Rate

**Figure 1:**
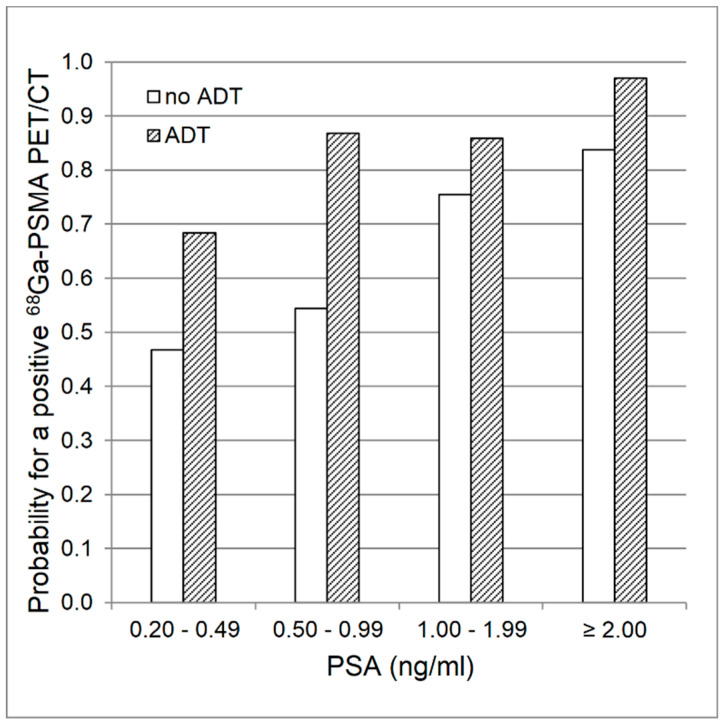
Logistic regression analysis. Estimated mean probability for a positive ^68^Ga-PSMA ligand PET/CT finding with regards to PSA values and presence of androgen deprivation therapy (ADT).

The localizations of tumor recurrence for both groups are shown in Table 3. Recurrence was more often detected in the group with ADT than in the control group (88.7% vs. 72.6%, *p* = 0.02) and was higher with increasing PSA-levels in both groups (ADT: 60.0% (PSA: 0.2 to < 0.5 ng/mL), 85.7% (0.5 to < 1.0 ng/mL), 92.9% (1.0 to < 2.0 ng/mL), 96.8% (2.0 to < 20.0 ng/mL); no ADT: 37.5% (0.2 to < 0.5 ng/mL), 63.6%(0.5 to < 1.0 ng/mL), 88.9% (1.0 to < 2.0 ng/mL), 79.4% (2.0 to < 20.0 ng/mL)). Due to the small sample sizes, we refrained from further statistical comparisons of the PSA-level subgroups. Within the ADT group, rates of positive PET/CT findings were slightly higher for CRPC as compared to HSPC (95.0% vs. 85.7%). Tumor detection rate tended to be lower in subjects with a Gleason score of 5 to 7 as compared to those with a higher Gleason score (8–10) (ADT: 83.9% (Gleason 5–7), 93.5% (8–10); *p* = 0.23; no ADT: 67.6% (5–7), 78.6% (8–10); *p* = 0.34; both groups: 75.4% (5–7), 86.4% (8–10); *p* = 0.12). Logistic regression analysis over both groups revealed, that log PSA (*p* < 0.001) and ADT (*p* = 0.02) were predictors of a positive PET/CT, whereas injected radioactivity per kg body weight, prior radiation therapy, and Gleason score were not. For log PSA, the probability of a positive ^68^Ga-PSMA I&T PET/CT finding increased with an odds ratio of 2.5 (95% confidence interval: 1.4–4.5) per unit, and for ADT, the odds ratio was 3.9 (95% confidence interval: 1.3–11.6) (Figure 1).

### 3.3. Quantitative Parameters

**Figure 2:**
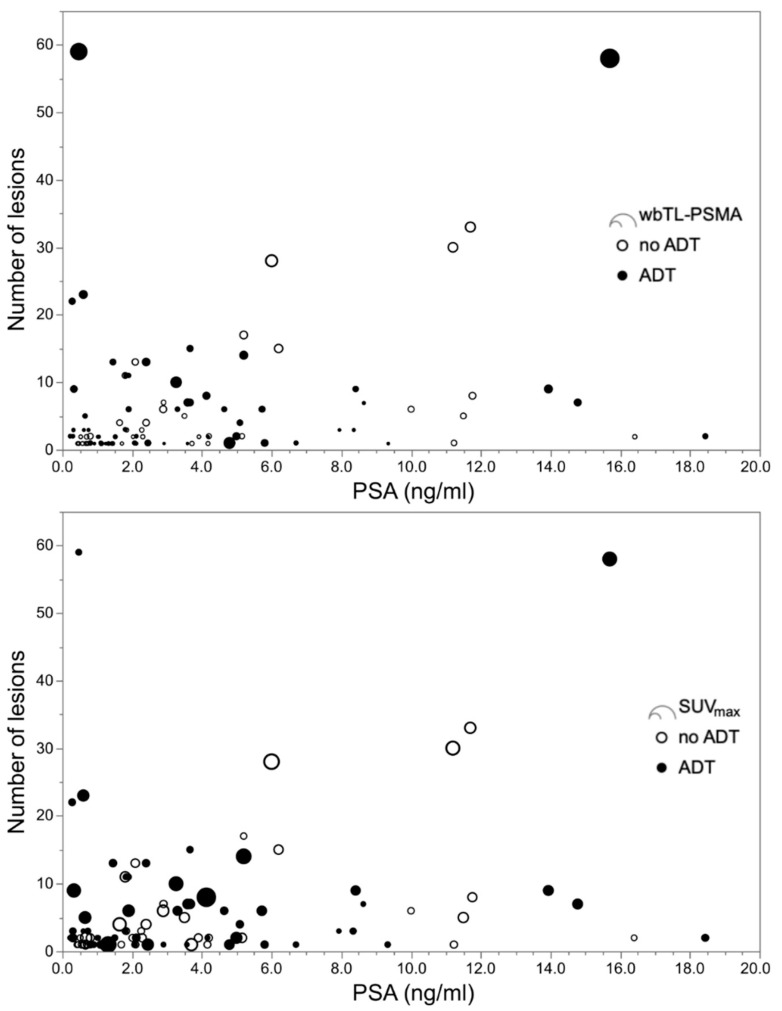
Plots with number of suspicious lesions over blood levels for prostate-specific antigen (PSA). Displayed are the results of ^68^Ga-PSMA I&T PET/CT derived parameters of 55 patients with and 45 patients without androgen deprivation therapy (ADT), and pathological imaging findings. The upper panel shows whole-body total lesion PSMA (wbTL-PSMA), the lower panel indicates the highest maximum standardized uptake value (SUV_max_) for each patient. Nine patients with ADT had a PSA value below 2.0 ng/mL, but ≥ 5 suspicious lesions, whereas this applied for only one patient without ADT.

All examined ^68^Ga-PSMA I&T PET/CT derived parameters, i.e., number of suspicious lesions, highest SUV_max_ per patient, wbTL-PSMA and wbPSMA-TV were significantly higher in the group with ADT than in the group without ADT (Table 4). In patients with ADT, correlations with PSA-levels were significant, though low (*σ* < 0.40) between PSA-values and SUV_max_, wbTL-PSMA, and wbPSMA-TV. The coefficient was slightly higher for wbTL-PSMA and wbPSMA-TV (*σ* = 0.52 and 0.51, respectively) in the group without ADT, where also number of lesions correlated with PSA-levels (*σ* = 0.50). Since these comparisons and correlations were substantially influenced by the number of negative cases, we excluded these patients and repeated the analysis. Thereafter, only wbTL-PSMA and wbPSMA-TV differed between groups and correlations between PSA levels and number of lesions (*σ* = 0.56), wbTL-PSMA (*σ* = 0.60), and wbPSMA-TV (*σ* = 0.58) were only present in patients without antiandrogen treatment (Table 4, Figure 2 and Supplementary Figure S2).

## 4. Discussion

This study was designed to directly compare the detection rate of ^68^Ga-PSMA ligand PET/CT in patients with recurrent PC, and with and without ADT, respectively. There are two main findings: first, the detection rate is higher in patients receiving ADT than in patients, who have comparable PSA-levels but do not undergo ADT. Second, the deviant detection rate is more likely related to the advanced disease stage of patients with therapy, than to a treatment induced effect on PSMA-expression.

The precise detection and localization of tumor recurrence is crucial for subsequent management in patients with rising PSA-levels after initial, curative treatment with RP. This comparison of patients with and without ADT revealed an overall higher detection rate in patients with ADT, which was also present in each subgroup according to the respective PSA-level. This leads to a higher mean probability for a pathological finding in ^68^Ga-PSMA ligand PET/CT in patients receiving antiandrogen treatment (Figure 1). This is in line with prior studies on PSMA ligand PET/CT gathering evidence for a higher detection rate in patients with ADT [10,12,13,14]. However, this is the first study comparing groups, which were balanced for relevant factors, such as PSA-levels, Gleason score and prior radiation treatment.

The detection rate of patients without treatment (72.6%) lies within the range (69.9%–76.7%) of prior studies using the same radioligand, ^68^Ga-PSMA I&T, though these rates refer to cohorts also containing patients with ADT [22,23]. As expected, we observed a rate for ^68^Ga-PSMA I&T PET/CT below the detection efficacy in studies using other PSMA ligands such as ^68^Ga-PSMA-HBED-CC (87.1%) or ^18^F-PSMA-1007 (78.0%) [13,14]. Although not balanced for PSA levels, these studies likewise reported a higher detection rate in patients with ADT (95.7% and 91.7%, respectively). This suggests that our finding is not limited to ^68^Ga-PSMA I&T and can be transferred to other radioligands targeting PSMA expression. As previously reported, tumor recurrence was localized more often in patients with a Gleason score ≥ 8 than in patients with a Gleason ≤ 7, albeit not reaching statistical significance [10,11,12,13,14]. This was also true for each group separately, indicating the lack of an interaction between Gleason score and ADT, which influences the detection rate.

There are two potential explanations for the higher detection rate in patients with ADT: a higher PSMA-expression induced by ADT in tumor cells and thus, a higher visibility of tumor lesions, or higher tumor burden related to an advanced disease stage of patients with ADT. Whereas the duration of ADT was neither associated with a positive or negative PET finding nor with higher wbTL-PSMA and wbPSMA-TV in the ADT group, we observed higher wbTL-PSMA and wbPSMA-TV and, albeit not significantly, more patients had visceral and bone metastases within the ADT group than in the group without treatment. Furthermore, both groups showed remarkably wide and predominantly overlapping ranges of SUV_max_. As depicted in Figure 2, high SUV_max_ values may occur in both groups in patients with high and low PSA-values, irrespective of the number of lesions. This suggests that the uptake is not primarily related to antihormonal treatment, but rather influenced by other factors such as the tumor cell proliferation of a specific lesion. During antihormonal treatment, PC typically develops androgen-resistance over time. Therefore, patients with CRPC have biologically different tumor lesions than HSPC patients within the ADT and the control group [24]. Subgroup evaluations showed a slightly higher detection rate and throughout higher quantitative imaging parameters in CRPC than HSPC patients and a continuum from CRPC over HSPC to PC without ADT (see Supplementary Table S1). However, a statistical comparison was not performed since PSA levels were unbalanced between groups, and biochemical and radiological surveillance (e.g., frequency of PSA level evaluation, baseline imaging) was not standardized in this retrospective cohort, potentially concealing a higher fraction of CRPC patients. This also precludes any conclusion on whether the castration state influences PSMA uptake, for which inconclusive findings have been presented so far [25,26]. Summarizing, a higher tumor burden (and/or more tumor lesions) and not a direct effect on PSMA-expression are likely the cause of better visibility and lead to a higher detection rate in patients with ADT.

These considerations refer to the rationale for antihormonal treatment. According to current guidelines [5], ADT can be considered in BCR in case of a short PSA doubling time < 6–12 months, at relapse, symptomatic progress, or distant metastasis. Due to therapy assignment, patients with ADT are highly likely to be in more advanced disease stages than patients without treatment. However, current clinical practice of patient surveillance, equally relies in both groups on PSA monitoring [27,28]. Whereas decreasing or low PSA-levels justify a wait-and-watch strategy in asymptomatic patients, increasing levels provoke action and lead to further diagnostics. Our data confirm previously reported correlations between PSA levels and tumor volume (i.e., wbTL-PSMA and wbPSMA-TV) in the group without ADT [20,21]. Though, the lack of correlation between PSA levels and tumor volume in patients with ADT challenges the reliability of PSA monitoring during antihormonal treatment. This is also reflected by the high numbers of patients with a PSA level < 2 ng/ml but more than five suspicious lesions (ADT: nine patients, no ADT: one patient; see Figure 2). We carefully reviewed the clinical charts to control for whether these patients had a (treatment-related) neuroendocrine prostate cancer, which would explain low PSA-values during advanced disease stages [29]. This was not the case in any of the patients at RP. However, prostate adenocarcinoma with neuroendocrine differentiation not only show low PSA-values, but also no or low PSMA expression [30], which conflicts with a moderate SUV_max_ in most of these patients (Figure 2).

According to these findings, withdrawal of ADT cannot be recommended before PSMA ligand PET/CT imaging, also not for patients with low PSA values. Considering the high detection rate in patients with PSA-levels <2 ng/mL and the number of patients with low PSA value and advanced disease stage, some patients might experience the risk of an accelerated tumor progression after treatment stop. Regarding tumor surveillance during ADT, PSMA ligand PET/CT seems to be superior towards the control of PSA blood levels in some cases. However, a broad clinical application is hampered due to the imbalance of costs and benefits.

This study has some limitations. Due to the retrospective analysis, the duration of ADT, biochemical surveillance and baseline imaging was not standardized so that included patients have a broad range of treatment duration and have biologically heterogeneous tumor lesions (i.e., HSPC and CRPC). Furthermore, we do not know the reason why treating physicians started ADT and which information were available to them at the time of treatment initiation. We assumed treatment decisions according to valid guidelines and paid attention, that selected covariates for the matching did not interfere with these recommendations. Likewise, the PSMA-derived tumor volume as measure for tumor burden might be affected by the actual PSMA expression. We controlled for this by comparing PSMA-derived tumor volume with morphologic, CT-based volume [31] and found strong correlations, which support our findings and make a bias unlikely (see Supplementary Figure S3). However, this study does not reveal the biological effect of short- and long-term ADT on PSMA expression, so that further studies also involving preclinical models [32] are warranted.

## 5. Conclusions

PSMA ligand PET/CT has a higher detection rate in patients with PSA relapse undergoing ADT than in subjects with comparable PSA-levels, who do not receive ADT. A better detection rate in patients with ADT is more likely related to an advanced biological disease stage with higher tumor burden. Considering the high detection rate in patients with PSA-levels < 2 ng/mL, the withdrawal of ADT before PSMA ligand PET/CT cannot be recommended.

## Figures and Tables

**Table 1:** Patient groups and covariates before and after Propensity score matching.

		All Patients			Matched Groups		
		ADT	No ADT	*p*	ADT	No ADT	*p*
**Patients**	**n**	65	334		62	62	
**PSA level**	**ng/mL**	2.1 (0.2–18.4)	0.8 (0.2–16.4)		2.0 (0.2–18.4)	2.2 (0.3–16.4)	
	**log PSA**	0.73 ± 1.20	−0.10 ± 0.96	<0.001 ^§^	0.67 ± 1.19	0.70 ± 1.07	0.880 ^§^
**Gleason score**	**5–7**	32	237	<0.001 ^#^	31	34	0.590 ^#^
	**8–10**	33	97		31	28	
**Radiation therapy**	**yes**	40	111	<0.001 ^#^	37	35	0.716 ^#^
	**no**	25	223		25	27	
**Injected activity**	**MBq/kg**	1.51 ± 0.32	1.52 ± 0.33	0.923 ^§^	1.50 ± 0.32	1.51 ± 0.31	0.855 ^§^

Abbreviations: ADT = androgen deprivation therapy, PSA = prostate-specific antigen; PSA levels are given for the time (< three months) prior to PET/CT, Gleason scores derived from prostatectomy specimens; *p* values refer to ^§^ students t and ^#^ chi-squared tests.

**Table 2:** Demographic and clinical characteristics of case and control groups.

		ADT	No ADT
**Age at primary diagnosis (years)**		62 (48–74)	64 (45–80)
**Time between RP and PET/CT (months)**		82 (1–239)	69 (1–240)
**Duration of ADT Treatment (months)**		36 (1–188)	n/a
**T stage ^†^**	**<3a**	30.5%	38.2%
	**≥3a**	69.5%	61.8%
**R status ^‡^**	**0**	67.5%	69.6%
	**1**	30.0%	28.3%
	**2**	2.5%	2.2%

Abbreviations: ADT = androgen deprivation therapy, RP = radical prostatectomy; T and R classification refer to pathological findings at prostatectomy; ^†^ available in 59 patients with and in 55 patients without ADT; ^‡^ available in 40 patients with and in 49 patients without ADT.

**Table 3:** Spreading of prostate cancer recurrence in ^68^Ga-PSMA I&T PET/CT.

Region	ADT	No ADT
LR only	4 (6.5%)	9 (14.5%)
LN metastases only	25 (40.3%)	20 (32.3%)
Bone metastases only	11 (17.7%)	5 (8.1%)
LR + LN metastases	4 (6.5%)	2 (3.2%)
LR + bone metastases	0 (0.0%)	2 (3.2%)
LN + bone metastases	7 (11.3%)	7 (11.3%)
LR + visceral metastases	1 (1.6%)	0 (0.0%)
LN + visceral metastases	1 (1.6%)	0 (0.0%)
LR + LN + bone metastases	1 (1.6%)	0 (0.0%)
LR + LN + visceral metastases	1 (1.6%)	0 (0.0%)
Total	55 (88.7%)	45 (72.6%)

Abbreviations: ADT = androgen deprivation therapy, LR = local recurrence, LN = lymph.

**Table 4:** Group comparison and correlational analysis of quantitative parameters in patients with and without ADT.

		Group Comparison			Correlation with PSA-Level			
		ADT	No ADT	*p* *	ADT	*p* °	No ADT	*p* °
**All patients** **n = 124**	**Number of lesions**	2.5 (0–59)	1.0 (0–33)	<0.01	0.24	0.06	0.50	<0.001
	**SUV_max_**	14.4 (0.0–181.1)	10.1 (0.0–107.1)	0.02	0.32	0.01	0.39	<0.01
	**wbTL-PSMA**	31.5 (0.0–1489.7)	5.4 (0.0–481.2)	<0.001	0.37	<0.01	0.52	<0.001
	**wbPSMA-TV**	3.7 (0.0–268.1)	1.0 (0.0–41.0)	<0.001	0.38	<0.01	0.51	<0.001
**Patients with positive PET/CT** **n = 100**	**Number of lesions**	3.0 (1–59)	2.0 (1–33)	0.15	0.07	0.60	0.56	<0.001
	**SUV_max_**	17.3 (5.1–181.1)	13.9 (4.4–107.1)	0.27	0.18	0.18	0.32	0.03
	**wbTL-PSMA**	39.5 (2.8–1489.7)	12.8 (1.3–481.2)	<0.01	0.24	0.08	0.60	<0.001
	**wbPSMA-TV**	4.9 (0.2–268.1)	1.6 (0.1–41.0)	<0.01	0.26	0.05	0.58	<0.001

Abbreviations: ADT = androgen deprivation therapy, PSA = prostate-specific antigen, SUV_max_ = maximum standardized uptake value, wbTL-PSMA = whole-body total lesion PSMA, wbPSMA-TV = whole-body PSMA total volume, *p* values refer to * Mann-Whitney U test and ° Spearman’s *σ*.

## Supplementary Materials

The following are available online at https://www.mdpi.com/2227-9059/8/11/511/s1., Figure S1: Patient flow, Figure S2: Plots with number of suspicious lesions over blood levels for prostate-specific antigen, Table S1: Quantitative parameters in patients with ADT according to the castration state, Figure S3: Plots with whole-body total lesion PSMA over whole-body CT derived tumor volume.
