# Supplementary material for: Detection Rate of 68Ga-PSMA Ligand PET/CT in Patients with Recurrent Prostate Cancer and Androgen Deprivation Therapy
Source: Biomedicines. 2020 Nov 18;8(11):511. doi: 10.3390/biomedicines8110511 (PMC7698713; doi:10.3390/biomedicines8110511)
Supplement: Supplementary file 1 [file biomedicines-08-00511-s001.pdf]

SUPPLEMENTAL DATA

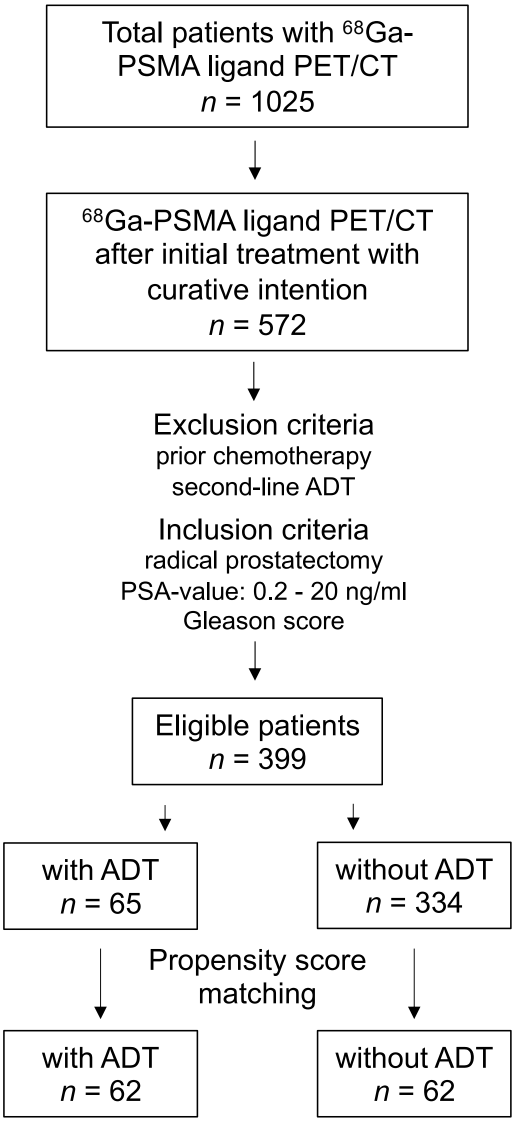

Figure S1. Patient flow. Abbreviations: PSA = prostate-specific antigen; ADT = androgen deprivation therapy.

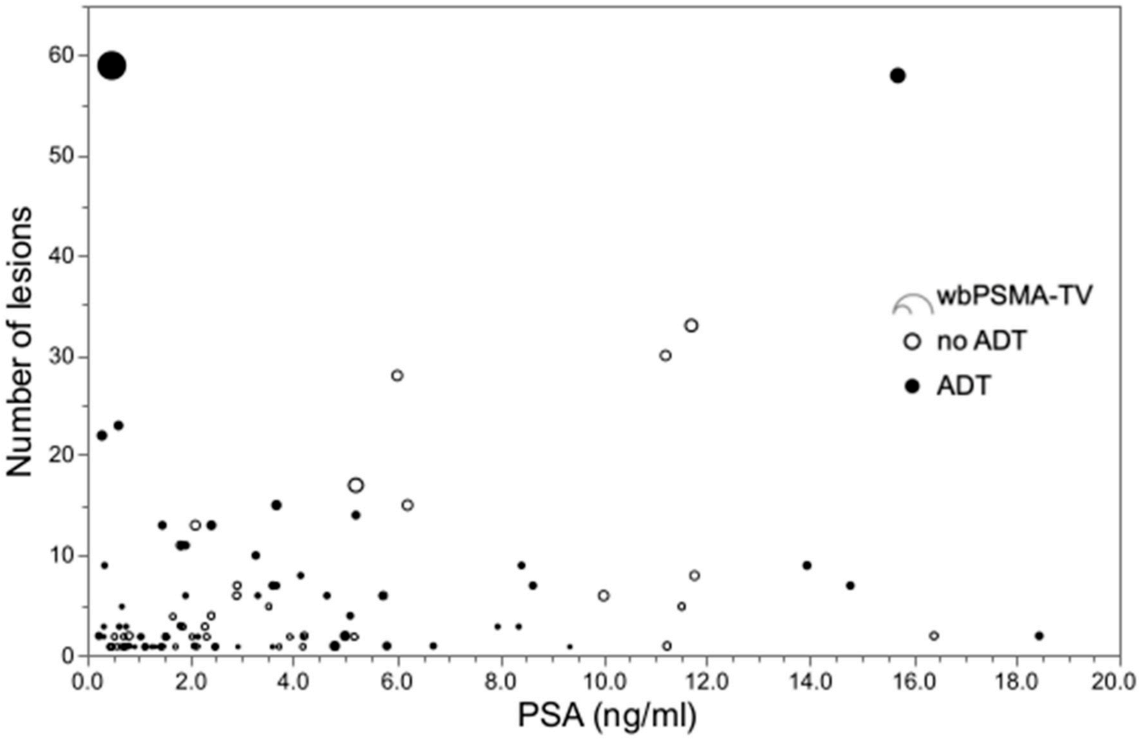

Figure S2. Plots with number of suspicious lesions over blood levels for prostate-specific antigen (PSA). Displayed are the results of 68Ga-PSMA I&T PET/CT derived whole-body tumor volume (wbPSMA-TV) of 55 patients with and 45 patients without androgen deprivation therapy (ADT), and pathological imaging findings.

Table S1. Quantitative parameters in patients with ADT according to the castration state

		ADT		no ADT n = 62
		**CRPC n = 20**	**HSPC n = 42**	
**All patients**	**PSA**	4.4 (0.61-5.7)	1.3 (0.2-18.44)	2.2 (0.3-16.4)
	**Number of lesions**	6.5 (0-58)	2.0 (0-59)	1.0 (0-33)
	**SUV_{max}**	28.8 (0-181.1)	11.2 (0-124.5)	10.1 (0.0-107.1)
	**wbTL-PSMA**	98.5 (0-1489.7)	16.3 (0-1152.4)	5.4 (0.0-481.2)
	**wbPSMA-TV**	7.3 (0-59.3)	2.2 (0-268.1)	1.0 (0.0-41.0)
**Patients with positive PET/CT**	**PSA**	4.6 (0.6-15.7)	1.5 (0.2-18.4)	2.3 (0.4-16.4)
	**Number of lesions**	7.0 (1-58)	2.0 (1-59)	2.0 (1-33)
	**SUV_{max}**	34.5 (6.3-181.1)	12.5 (5.1-124.5)	13.9 (4.4-107.1)
	**wbTL-PSMA**	106.7 (7.0-1489.7)	23.0 (2.8-1152.4)	12.8 (1.3-481.2)
	**wbPSMA-TV**	8.1 (1.8-59.3)	2.9 (0.2-268.1)	1.6 (0.1-41.0)

Abbreviations: ADT = androgen deprivation therapy, CRPC = castration-resistant prostate cancer, HSPC = hormone-sensitive prostate cancer, PSA = prostate-specific antigen, SUV_{max} = maximum standardized uptake value, wbTL-PSMA = whole-body total lesion PSMA, wbPSMA-TV = whole-body PSMA total volume, *p* values refer to *Mann-Whitney U test and °Spearman's σ.

CT-derived tumor volume. To estimate the morphologic tumor volume, we measured each suspected pathological lesion in CT images by using modified RECIST 1.1 criteria [1]. First, long-axis and short-axis diameter were measured for each lesion (local recurrence, lymph node and organ metastases) at the slice of the maximum diameter and their product was calculated (CT_{VOL}). We then summarized the area of all lesions per patient to obtain a whole-body CT-based tumor volume ($wbCT_{VOL}$). Due to insufficient sensitivity of CT in bone metastases, CT_{VOL} was not assessed in bone metastases.

Comparison of CT- and 68Ga-PSMA-PET/CT derived tumor volume. We used Spearman`s rank correlation coefficient to compare CT- and PSMA-based tumor volume in all patients without bone metastases. Both measures correlated strongly with $wbCT_{VOL}$ though correlation was better for wbTL-PSMA than for wbPSMA-TV overall (wbTL-PSMA: $\sigma = 0.87$, $p<0.0001$; wbPSMA-TV: $\sigma = 0.81$, $p<0.0001$). This was also true within each patient group (wbTL-PSMA, ADT: $\sigma = 0.85$, $p<0.0001$; wbTL-PSMA, no ADT: $\sigma = 0.92$, $p<0.0001$; wbPSMA-TV, ADT: $\sigma = 0.78$, $p<0.0001$; wbPSMA-TV, no ADT: $\sigma = 0.82$, $p<0.0001$) (Supplemental Figure S3).

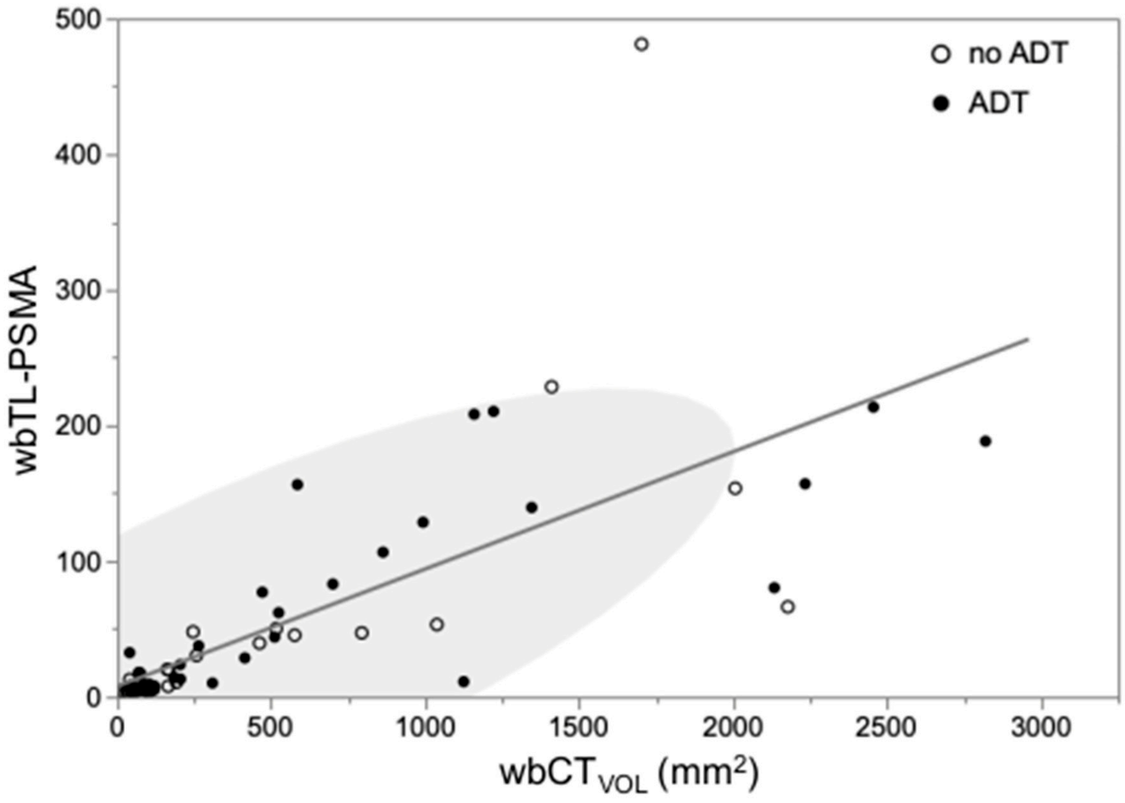

Figure S3. Plots with whole-body total lesion PSMA (wbTL-PSMA) over whole-body CT derived tumor volume (wbCT$_{VOL}$). Displayed are the parameters of patients with pathological imaging findings with and without androgen deprivation therapy (ADT), but only for patients without bone metastases (ADT: n = 35; no ADT: n = 31).

REFERENCES

1. Schwartz LH, Litiere S, de Vries E, Ford R, Gwyther S, Mandrekar S et al. RECIST 1.1-Update and clarification: From the RECIST committee. Eur J Cancer. 2016;62:132-7.
